# Delaying Shoulder Motion and Strengthening and Increasing Achilles Allograft Thickness for Glenoid Resurfacing Did Not Improve the Outcome for a 30-Year-Old Patient with Postarthroscopic Glenohumeral Chondrolysis

**DOI:** 10.1155/2014/517801

**Published:** 2014-12-14

**Authors:** John G. Skedros, Tanner R. Henrie, Chad S. Mears

**Affiliations:** ^1^Department of Orthopaedic Surgery, The University of Utah, Salt Lake City, UT 84108, USA; ^2^Utah Orthopaedic Specialists, Salt Lake City, UT 84107, USA; ^3^Intermountain Medical Center, Salt Lake City, UT 84157, USA

## Abstract

Although interposition soft-tissue (biologic) resurfacing of the glenoid with humeral hemiarthroplasty has been considered an option for end-stage glenohumeral arthritis, the results of this procedure are highly unsatisfactory in patients less than 40 years old. Achilles tendon allograft is popular for glenoid resurfacing because it can be made robust by folding it. But one reason that the procedure might fail in younger patients is that the graft is not initially thick enough for the young active patient. Most authors report folding the graft only once to achieve two-layer thickness. We report the case of a 30-year-old male who had postarthroscopic glenohumeral chondrolysis that was treated with Achilles tendon allograft resurfacing of the glenoid and humeral hemiarthroplasty. An important aspect of our case is that the tendon was folded so that it was 50–100% thicker than most allograft constructs reported previously. We also used additional measures to enhance allograft resiliency and bone incorporation: (1) multiple nonresorbable sutures to attach the adjacent graft layers, (2) additional resorbable suture anchors and nonresorbable sutures in order to more robustly secure the graft to the glenoid, and (3) delaying postoperative motion and strengthening. However, despite these additional measures, our patient did not have an improved outcome.

## 1. Introduction

Interposition biologic (soft-tissue) resurfacing of the glenoid with stemmed humeral hemiarthroplasty (i.e., conventional hemiarthroplasty) or humeral head resurfacing is an option for younger patients with end-stage glenohumeral arthritis [1–3]. Methods for glenoid resurfacing include autogenous anterior glenohumeral capsular tissue, autogenous fascia lata, meniscus allograft, Achilles tendon allograft, and human dermal matrix scaffold allograft [1, 3–9]. Achilles tendon allograft is becoming popular for glenoid resurfacing because it can be made robust by folding it. Krishnan et al. [5] advocated folding the tendon to achieve three- to four-layer thickness. They reported on 34 patients (mean age 51) who had soft-tissue resurfacing and conventional humeral hemiarthroplasty. Eighteen of these patients were treated with Achilles tendon and all of these 18 patients had satisfactory results (Table 1).

However, in younger patients, the success of soft-tissue resurfacing of the glenoid is reported as highly unsatisfactory [3, 4, 9]. Elhassan et al. [4] reported on 13 patients with an average age of 34 years (range: 18–49) who were treated with soft-tissue glenoid resurfacing and conventional hemiarthroplasty. In 11 of these 13 patients this was done with Achilles tendon allograft that was folded over once, achieving two-layer thickness. Besides using a thinner graft, the methods employed to attach the graft resemble those of Krishnan et al. [5] (Table 1). However, 10 (77%) of patients reported by Elhassan et al. did poorly, being converted to a total shoulder arthroplasty (TSA) at a mean of 14 months after surgery.

Muh et al. [3] also reported unsatisfactory outcomes (38% converted to TSA) in their relatively younger patients (*n* = 16; mean 36 years old) who had conventional hemiarthroplasty with soft-tissue resurfacing of the glenoid. Of the nine patients who had an Achilles allograft, four (44%) were converted to TSA at an average time of approximately 3 years later. In their series the Achilles tendon was folded over once (Table 1).

We report the case of a 30-year-old male who had intra-articular pain-pump catheter associated (PPCA) postarthroscopic glenohumeral chondrolysis (PAGCL) that was treated with Achilles tendon allograft resurfacing of the glenoid and conventional humeral hemiarthroplasty. An important aspect of our case is that the tendon was folded so that it was 25% thicker than the thickest allograft construct (8 mm) used by Krishnan et al. [5]. We also used additional measures to increase allograft resiliency: (1) multiple nonresorbable sutures to attach the adjacent layers of the graft, (2) additional resorbable suture anchors and nonresorbable sutures in order to more robustly secure the graft to the glenoid, and (3) delaying postoperative motion and strengthening (Table 1). These additional measures reflected our presumption that they would prolong graft resiliency during the process of biological assimilation (i.e., biological attachment of the graft to the bone and its reconstitution with host tissue) [2, 10, 11]. However, even with these modifications, our patient had a poor result, being converted to a TSA two years later.

## 2. Case Report

This healthy right-hand-dominant 30-year-old male (BMI = 33) presented to our clinic with a chief complaint of left shoulder stiffness and pain that had progressively increased to be severe over the prior two years. He also reported a grinding sensation with minimal shoulder motion. The patient's first left shoulder surgery, performed 3.5 years earlier (August 2004), was an arthroscopic anterior and posterior capsular shift for atraumatic instability. Over the next two years he developed significant radiographic narrowing of the glenohumeral joint. In October 2007 a diagnostic arthroscopy revealed end-stage chondrosis of the glenohumeral joint. At his initial surgery a pain-pump catheter was placed into the glenohumeral joint and delivered (high-flow) bupivacaine for pain control [12].

Three years later (November 2010) he had a conventional humeral hemiarthroplasty and resurfacing of the glenoid using an Achilles tendon allograft. The surgery was performed by John G. Skedros generally in accordance with the description of Krishnan et al. [5]; however, the Achilles tendon selected was relatively large/robust and was folded until it was four layers thick, resulting in 10.5 mm thickness (Krishnan et al. [5] reported 5–8 mm). We speculated that thicker tissue would allow additional time for deeper tissue layers to become incorporated into the bone surface before erosion of the superficial layers stabilized. This hypothesis seemed to be supported by findings of Krishnan et al. [5] showing that glenoid erosion averaged 7 mm, apparently becoming stable after several years.

Additional measures taken to further augment graft resiliency included the following: (1) adjacent layers of the Achilles tendon were sewn together with multiple nonresorbable sutures (number 2-0 FiberWire; Arthrex, Naples, FL, USA), (2) six resorbable suture anchors (two more than those used in prior studies; Table 1), each double loaded with nonresorbable suture (Lupine anchors with number 2 Orthocord suture; DePuy Mitek, Raynham, NJ, USA), were inserted equidistantly on the glenoid, and (3) number 2 sutures were also passed through drill holes around the glenoid rim [5]. These enhancements also reflected our hypothesis that perhaps up to three years would be required for biological assimilation at the graft-bone interface as shown for anterior cruciate ligament (ACL) allografts [10, 13].

Our postoperative protocol also delayed all motion and strength activities when compared to prior protocols (Table 1). When strengthening was begun at 16 weeks after surgery, isometric exercises emphasized avoiding shoulder subluxations [14].

By four months after surgery the patient stated that he was feeling “better than ever.” However, the pain increased to a high level by eight months after surgery. Radiographic joint space narrowing was progressive over the following two years.

**Figure 1:**
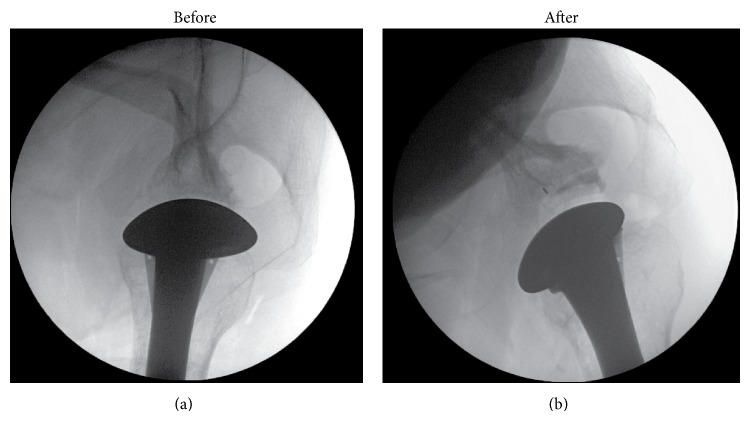
Intraoperative axillary-lateral radiographs of the hemiarthroplasty with glenoid soft-tissue resurfacing: (a) prior to conversion to the TSA and (b) after conversion to the TSA.

Two years after the biologic resurfacing procedure, the patient underwent conversion to a TSA (Figure 1). Operative findings revealed that the allograft had completely disintegrated. Revision included placing a smaller humeral head and a pegged polyethylene glenoid component with bone cement. At follow-up 2.5 years later he was very satisfied with his final result.

## 3. Discussion

We had hoped that the measures taken to physically enhance the resiliency and fixation of our patient's Achilles allograft, in addition to slowly progressing shoulder use, would help curb the rate of graft erosion, yielding the good outcomes reported by Krishnan et al. [5]. But our patient was converted to a TSA in a timeframe resembling patients that were also converted to a TSA in Elhassan et al. [4]. The obvious similarity between our patient and those of Elhassan et al. [4] is that they were relatively young—about 20 years younger than the average age of patients of Krishnan et al. [5].

In a critique of Krishnan et al. [5], Matsen [14] enumerated several considerations that likely reflect, or influence, the rate of glenoid graft erosion as follows.

1. Durability: the average radiographic joint space diminished from 2.9 mm immediately after surgery to 1.3 mm at the time of the most recent follow-up. It is not clear whether the residual radiographic joint space was occupied by the original interpositional material or by new tissue ingrowth.
2. Fixation to bone: no data were provided on the degree to which the grafts healed and remained fixed to the subjacent bone.
3. Load distribution: glenoid erosion averaged 7.2 mm, apparently becoming stable after several years (Krishnan et al. report five years). It may be that this erosion takes place until the load is evenly distributed across the surface of the glenoid bone.
4. Intrinsic stability: three of the thirty-six shoulders demonstrated postoperative instability. It is not clear whether this procedure reestablishes the glenoid concavity or whether these cases of instability were due to lack of effective concavity or due to other causes.

We suggest that in patients with PPCA PAGCL their glenohumeral inflammation is much more unfavorable to the biological assimilation/incorporation of an allograft when compared to patients with typical degenerative arthritis. This idea seems to be supported by several studies where 50–100% of the PAGCL patients (some from thermal capsulorrhaphy without use of a pain-pump) had persistent high pain despite having hemiarthroplasty or TSA [12, 15, 16] (reviewed by Busfield and Romero [17]). If this interpretation is correct, then increased suture density in our patient's graft and the presence of allograft tissue itself added antigenic/inflammatory components that promoted the already adverse/inflammatory environment [2]. This seems supported by the long-term outcome of hemiarthroplasties in younger patients at the Mayo Clinic that is much better than hemiarthroplasties with interposition allograft [9, 18]. It is encouraging that when PPCA PAGCL patients had an arthroplasty they had better outcomes when TSA was the initial arthroplasty. Levy et al. [19] reported on 11 patients with PAGCL (average age 39 years, range: 16–64) and “at least nine” of these had PPCA PAGCL. All 11 were treated with TSA at a mean of 26 months (range: 8–51 months) after arthroscopy. Seven patients had excellent results, three good, and one satisfactory. Hasan and Fleckenstein [20] also report generally good results in the PPCA PAGCL patients that were treated with TSA as the initial arthroplasty.

In order to reduce the potential for exacerbating the antigenic/inflammatory glenohumeral environment when biologically resurfacing the glenoid, it seems reasonable to consider using materials that have reduced antigenicity. Xenogeneic and allogeneic cellular antigens are, by definition, recognized as foreign by the host and therefore induce an inflammatory response or an immune-mediated rejection of the tissue [21, 22]. Because of this, decellularization of these materials is commonly done to remove all cellular and nuclear material while minimizing any adverse effect on the composition, biological activity, and mechanical integrity of the remaining extracellular matrix [21]. In this context a bulk Achilles tendon allograft like that used in our patient might be less desirable than newer decellularized materials for use in glenoid resurfacing regardless of whether or not patients have PPCA PAGCL. But enthusiasm for this possibility is greatly diluted by the results of the recent study of Strauss et al. [9] who studied the outcomes of biological resurfacing of the glenoid using a lateral meniscus allograft or human acellular dermal tissue matrix (45 total patients, mean age 42 years). They reported that the lateral meniscal cohort had a failure rate of 45.2% at a mean time of 3.4 years and the human acellular dermal tissue matrix cohort had a 70.0% failure rate at a mean time of 2.2 years. With the exception of possibly one patient, the remaining 44 patients in their study did not have PPCA PAGCL. Muh et al. [3] also reported high failure rates (44% at a mean of three years postoperatively) in seven of their total of 16 patients that had glenoid resurfacing with either Achilles tendon allograft (*n* = 9) or human acellular dermal tissue matrix (*n* = 7) (none of these patients had PPCA PAGCL). Of the seven patients that failed early, three had glenoid resurfacing with human acellular dermal tissue matrix. All of these seven patients were converted to a TSA and they had, on average, worse postoperative visual analogue scale (VAS) pain scores than the patients who did not require revision to TSA (8.4 versus 3.8). Patients with PPCA PAGCL would likely have even worse outcomes because this is a distinct clinical entity that has a poor track record when treated with methods that are less than a TSA, which have included arthroscopic debridement with chondroplasty, arthroscopic capsular release, arthroscopic microfracture to stimulate fibrocartilage formation, meniscal allograft, other tissues interposition, osteoarticular allograft, humeral head prosthetic resurfacing, and humeral hemiarthroplasty [12, 17, 19, 20, 23–25]. Very good to excellent outcomes with any arthroplasty technique are also less likely achieved in PPCA PAGCL patients.

## 4. Conclusion

Our patient did not have an improved outcome even though we (1) more robustly attached an Achilles tendon that was also folded to achieve a thickness 25% greater than the thickest graft used by Krishnan et al. [5] and (2) slowly progressed motion and strengthening. We speculate that the allograft might add an antigenic/inflammatory component that enhances failure of glenoid interpositional soft-tissue in patients with PPCA PAGCL.

## Figures and Tables

**Table 1:** Data from the present report and selected studies of patients with glenohumeral arthritis treated with soft-tissue resurfacing of the glenoid with humeral hemiarthroplasty or humeral head resurfacing.

First author, year	Mean patient age (range)^*^	Number with Achilles allograft	% with Achilles revised to TSA	Graft thickness	Number of suture anchors used on glenoid surface	Peripheral sutures used?	Time to passive motion	Time to active-assisted motion	Time to active motion	Time to strengthening (recreational activities)
Krishnan, 2007 [5]	51 (30–75)	18	0%	5–8 mm (folded to 3-4 layers)	4	Yes (4 transosseous Bankart type)	Immediate	NR	4 weeks	8 weeks (NR)

Elhassan, 2009 [4]	34 (18–49)	10	77%	Folded once (2 layers)	4	Yes	Immediate	4 weeks	4 weeks	12 weeks (16 weeks)

Muh, 2014 [3]	36	9	44%	Folded once	4	Yes	2–6 weeks	6 weeks	4–8 weeks	12 weeks^†^
	(14–45)			(2 layers)^†^		(running mattress)	[Two different protocols used]			(NR)

Current report	30	1	100%	10.5 mm (4 layers)	6	Yes (similar to Krishnan)	4 weeks	8 weeks	12 weeks	16 weeks (36 weeks)

^*^Average ages are from the entire sample of patients described in each study.

NR: not reported; ^†^personal communication from Dr. Reuben Gobezie.

Besides our case, these other studies did not have patients with intra-articular pain-pump catheter associated (PPCA) postarthroscopic glenohumeral chondrolysis (PAGCL). (For Muh et al. [3] this information was provided via personal communication from Dr. Reuben Gobezie.)
